# Conserved Domains in Variable Surface Lipoproteins A-G of *Mycoplasma hyorhinis* May Serve as Probable Multi-Epitope Candidate Vaccine: Computational Reverse Vaccinology Approach

**DOI:** 10.3390/vetsci10090557

**Published:** 2023-09-05

**Authors:** Muhammad Zubair, Jia Wang, Yanfei Yu, Muhammad Asif Rasheed, Muhammad Faisal, Ali Sobhy Dawood, Muhammad Ashraf, Guoqing Shao, Zhixin Feng, Qiyan Xiong

**Affiliations:** 1 Key Laboratory of Veterinary Biological Engineering and Technology, Ministry of Agriculture, Institute of Veterinary Medicine, Jiangsu Academy of Agricultural Sciences, Nanjing 210000, China; 20200019@jaas.ac.cn (M.Z.); 20130986@jaas.ac.cn (J.W.); 20160065@jaas.ac.cn (Y.Y.); 19880016@jaas.ac.cn (G.S.); 20070022@jaas.ac.cn (Z.F.); 2 GuoTai (Taizhou) Center of Technology Innovation for Veterinary Biologicals, Taizhou 225300, China; 3 School of Food and Biological Engineering, Jiangsu University, Zhenjiang 212013, China; 4 College of Veterinary Medicine, Nanjing Agricultural University, Nanjing 210095, China; 5 Department of Biosciences, COMSATS University Islamabad, Sahiwal Campus, Islamabad 45550, Pakistan; asif.rasheed@cuisahiwal.edu.pk; 6 Division of Hematology, Department of Medicine, The Ohio State University College of Medicine, The Ohio State University Comprehensive Cancer Center, Columbus, OH 43210, USA; muhammad.faisal@osumc.edu; 7 The State Key Laboratory of Agricultural Microbiology, Department of Preventive Veterinary Medicine, Huazhong Agricultural University, Wuhan 430070, China; ali.dawood@vet.usc.edu.eg; 8 Faculty of Veterinary Medicine, University of Sadat City, Sadat City 32897, Egypt; 9 Institute of Microbiology, University of Agriculture Faisalabad, Faisalabad 37000, Pakistan; mashraf@uaf.edu.pk; 10 School of Life Sciences, Jiangsu University, Zhenjiang 212013, China

**Keywords:** conserved domains, immunoinformatics, multiepitope-based vaccine, *M. hyorhinis*, reverse vaccinology, variable surface lipoproteins

## Abstract

**Simple Summary:**

In this study, we designed a multi-epitope vaccine candidate for *M. hyorhinis*. This is the first study using conserved regions of seven members of Vlps and has shown an effective approach toward vaccine development. The proposed vaccine candidate is highly stable and soluble. It elicits an impressive immune response and can be regarded as a hypothetical vaccine candidate in the future. Further laboratory tests and animal inoculation are required to validate vaccine efficacy and safety to combat swine infections.

**Abstract:**

*Mycoplasma hyorhinis* (*M. hyorhinis*) is responsible for infections in the swine population. Such infections are usually cured by using antimicrobials and lead to develop resistance. Until now, there has been no effective vaccine to eradicate the disease. This study used conserved domains found in seven members of the variable lipoprotein (VlpA-G) family in order to design a multi-epitope candidate vaccine (MEV) against *M. hyorhinis*. The immunoinformatics approach was followed to predict epitopes, and a vaccine construct consisting of an adjuvant, two B cell epitopes, two HTL epitopes, and one CTL epitope was designed. The suitability of the vaccine construct was identified by its non-allergen, non-toxic, and antigenic nature. A molecular dynamic simulation was executed to assess the stability of the TLR2 docked structure. An immune simulation showed a high immune response toward the antigen. The protein sequence was reverse-translated, and codons were optimized to gain a high expression level in *E. coli*. The proposed vaccine construct may be a candidate for a multi-epitope vaccine. Experimental validation is required in future to test the safety and efficacy of the hypothetical candidate vaccine.

## 1. Introduction

Several species of the genus Mycoplasma infect and produce disease in pigs. Harboring respiratory tracts include *M. hyorhinis, M. hyopneumoniae,* and *M. flocculare* [1]. *M. hyorhinis* resides and colonizes in the respiratory tract of healthy and infected pigs and colonizes most likely due to stress as the mechanisms are not yet well known. The disease is clinically manifested through polyserositis, conjunctivitis, otitis, arthritis, and lameness [2]. Moreover, *M. hyorhinis* is also known to be involved in human cancer. The pathogen has been reported to augment the cancer cell migration, invasion, and metastasis significantly [3], and anti-P37 antibody was found to inhibit the cancer cell migration.

*Mycoplasma hyorhinis* belongs to the class mollicutes, lacks cell walls, and has been successfully adopted in host cell environments. Cell membrane surface variations are one of their adaptation strategies to the different host cell environments. These surface variations are due to variable surface lipoproteins (Vlps) possessing a high-frequency phase and size variation resulting in structural repetition at the C-terminal region [4]. This family of Vlps comprises seven members: VlpA, VlpB, VlpC, VlpD, VlpE, VlpF, and VlpG. All have been found to play significant roles as cytoadhesins as well as interacting with the host cell plasminogen and extracellular matrix (ECM); hence, they are considered to be important for persistent infections of *M. hyorhinis* [5,6].

Previously, Vlp-ELISA was developed by using unique repeated units at region III of VlpA-G in our laboratory. A total of 247 clinical samples from experimentally infected pigs were tested. The coefficients of variation for the inter-assay and intra-assay were below 10%. The established method did not show any cross-reactivity with other common pathogens infecting pigs and showed up to marked specificity and stability (article published in Chinese). In another study, ELISA was developed based on the chimeric VlpA-G recombinant polypeptide by in vitro synthesizing the Vlp coding genes of *M. hyorhinis*. A remarkable diagnostic and analytical specificity was detected against the pigs inoculated with *M. hyorhinis, M. hyosinoviae, M. hyopneumoniae,* and *M. floccularae* [7]. This shows that Vlps are important antigens of *M. hyorhinis*. This is not surprising that variable proteins also possess conserved domains, i.e., variant surface glycoprotein (VSG) of *Trypnosoma* sp. [8]. Conserved region IR6 in the Variable antigen VlsE of *B. burgdorferi* was supposed to act as a protective antigen [9]. Another study revealed that the conserved regions in variable *Mycoplasma mycoides* subsp. *caprii* lipoproteins stimulate the immune response and may act as immunogenic and protective antigens [10].

Inactivated vaccines are being used to prevent infections. These vaccines include the Boehringer Ingelheim Animal Health’s (BIAH) MHR vaccine and Ingelvac^®^ MycoMAX (Boehringer Ingelheim, Canada Ltd., Burlington, ON, USA) [11]. Another inactivated vaccine combined with circovirus type 2 was developed and showed a significant reduction in infections caused by *M. hyorhinis* and swine circovirus type 2 [12]. Although inactivated vaccines help reduce the lesions, they are not effective enough to control the infections at the desired level. Inactivated vaccines are formulated and administered without prior knowledge of mechanisms and the ability of immune activation [13]. The computational reverse vaccinology approach provided us a better way to predict the safety and efficacy of the proposed candidate vaccine prior to experimental trials. This approach has made it easier to screen the limited number of protective antigens from the whole genome [14]. There is still no subunit or multi-epitope vaccine that can be more reliable and effective against *M. hyorhinis*. 

To meet the limitations of inactivated vaccines, immunoinformatics is an extensively utilized approach to develop peptide vaccines against several bacterial pathogens, including *Mycobacterium tuberculosis*, *Mycoplasma pneumonia*, *Mycobacteroides abscessus*, *Helicobacter pylori*, *Campylobacter jejuni,* and many others [15,16,17,18,19]. Using the computational biology tools has provided the unequivocal route for selecting defined vaccine candidates and paved the way toward better vaccine production [20]. 

The objective of the current study is to use computational biology tools to identify and combine the conserved regions in all seven Vlp members and to test the efficacy of a multi-epitope vaccine construct as a candidate vaccine against *M. hyorhinis* infections.

## 2. Materials and Methods

### 2.1. Sequences and Alignment and Prediction of Epitopes

Sequences in the representative strain of *M. hyorhinis* (ATCC 17981) were obtained from NCBI protein database http://www.ncbi.nlm.nih.gov/protein/ (accessed on 30 July 2022) in FASTA format and multiple sequence alignment was conducted in order to find the conserved regions at N-terminal of each sequence. The IEDB server (http://tools.iedb.org/bcell/ (accessed on 30 July 2022)) was used to identify the B cell epitopes. BepiPred-2.0 (http://www.cbs.dtu.dk/services/BepiPred/ (accessed on 30 July 2022)) was used for further confirmation of B cell epitopes using antigenic sequence [21]. Emini Surface Accessibility Prediction tool, Kolaskar, and Tongaonker Antigenicity method were used to identify the antigenic sites, Karplus and Schulz’s flexibility to predict the flexibility, and The Parker Hydrophilicity Prediction tool was used to identify the hydrophilic regions within the conserved sequence.

NetCTL 1.2 (http://www.cbs.dtu.dk/services/NetCTL/ (accessed on 30 July 2022)) was used to predict CTL epitopes [22]. Epitopes were predicted based on three aspects, including binding affinity (threshold = 0.05), cleavage (threshold = 0.15), and TAP (Transporter Associated with Antigen Processing) (threshold = 0.75). The HTL epitopes were predicted using the IEDB MHC II server (http://tools.iedb.org/mhcii/ (accessed on 30 July 2022)) [23]. Swine Leukocyte Antigen (SLA) Alleles were randomly selected, and affinity was shown on percentile rank. Epitopes with low percentile rank have higher affinity. The IFN-gamma (http://crdd.osdd.net/raghava/ifnepitope/scan.php (accessed on 30 July 2022)) epitope server was used to predict interferon-gamma-inducing epitopes. Positive value meets the criteria for the construction of multi-epitope vaccine.

### 2.2. Construction of Multi-Epitope Vaccine

A candidate vaccine construct consisting of two B cell epitopes, two HTL epitopes, and one CTL epitope was combined using linkers, and an adjuvant β-Defensin was added to enhance the immunity. A vaccine construct with a length of 138 amino acids was designed. 

### 2.3. Prediction of Antigenicity

Antigenicity was measured using ANTIGENpro (http://scratch.proteomics.ics.uci.edu/ (accessed on 30 July 2022)) and VaxiJen v2.0 (http://www.ddg-pharmfac.net/vaxijen/VaxiJen/VaxiJen.html (accessed on 30 July 2022)). AllergenFP (https://ddg-pharmfac.net/AllergenFP/) was used to predict allergenicity of vaccine construct. ToxinPred server (https://webs.iiitd.edu.in/raghava/toxinpred/multi_submit.php (accessed on 30 July 2022)) was used to determine the toxicity of the vaccine construct.

### 2.4. Physicochemical Properties

The Expasy Protparam webserver (https://web.expasy.org/protparam/ (accessed on 30 July 2022)) was used for the prediction of the entire physicochemical properties of the vaccine construct. Solubility was determined using Protein–Sol server (https://protein-sol.manchester.ac.uk/ (accessed on 30 July 2022)).

### 2.5. Prediction of Secondary Structure

Secondary structure was predicted by using PRISPRED (http://bioinf.cs.ucl.ac.uk/psipred/ (accessed on 30 July 2022)) while RaptorX Property (http://raptorx.uchicago.edu/StructurePropertyPred/predict/ (accessed on 30 July 2022)) was used for secondary structure prediction using algorithms along with identification of solvent properties [17].

### 2.6. Prediction and Validation of Tertiary Structure

A 3-D modeled structure was obtained from I-TASSER online server (https://zhanggroup.org/I-TASSER/ (accessed on 30 July 2022)). Structural refinement was conducted using the GalaxyREFINE database (http://galaxy.seoklab.org/ (accessed on 30 July 2022)) [24]. Ramachandran Plot server (https://zlab.umassmed.edu/bu/rama/ (accessed on 30 July 2022)) was used for the verification of 3-D structure. Another method for validating the structure was based on z score using ProSA-web server (https://prosa.services.came.sbg.ac.at/prosa.php (accessed on 30 July 2022)).

### 2.7. Prediction of Discontinuous Epitopes

Structural-based computational analysis can determine the linear and discontinuous B cell epitopes. About 90% of the structure consists of discontinuous epitopes synthesized by the protein sequence in the pathogen. Ellipro web tool (http://tools.iedb.org/ellipro/ (accessed on 30 July 2022)) was applied with all parameters set as default and a 3D model of the vaccine construct was used as input. This tool uses an algorithm for estimating protein shape, protrusion index (PI) of the residues, and clustering. 

### 2.8. Molecular Docking

Pdb for TLR2 (id: Q59HI8) was retrieved from the Protein Databank (https://www.rcsb.org/). Docking was performed by using HADDOCK server (https://wenmr.science.uu.nl/prodigy/ (accessed on 30 July 2022)) [25].

### 2.9. Molecular Dynamic Simulation

iMODs (http://imods.chaconlab.org/ (accessed on 30 July 2022)) server was used for the molecular simulation of multi-epitope vaccine candidate [26]. 

### 2.10. Immune Simulation

C-IMMSIM (C language version of the IMMune system SIMulator) (https://150.146.2.1/C-IMMSIM/index.php (accessed on 30 July 2022)) was used to obtain the immune profile of the vaccine candidate.

### 2.11. Codon Optimization and In Silico Cloning

The Java Codon Adaption Tool (JCat) (http://www.jcat.de/ (accessed on 30 July 2022)) web server was used for codon optimization, and SnapGene trail user (https://www.snapgene.com/try-snapgene/) was used to clone the gene sequence into *E. coli* plasmid pET-30a (+) vector

## 3. Results

### 3.1. Multiple Sequence Alignment

**Figure 1:**
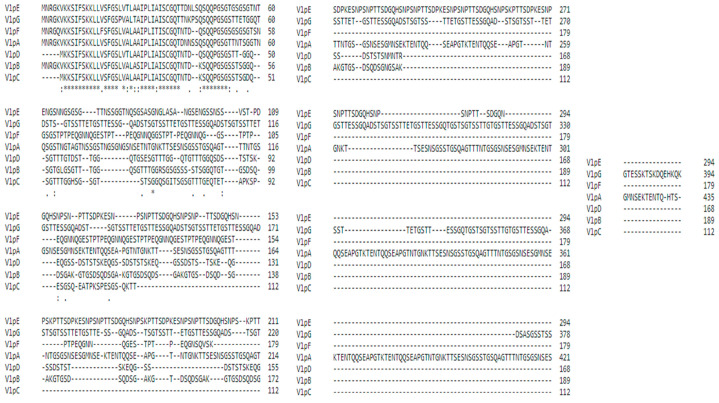
Multiple sequence alignment of seven members of Vlp shows the conserved domains at N terminal region. The asterisks represent the conserved regions in the multiple sequence alignment.

Sequences of all seven members of Vlps were retrieved from NCBI. These sequences were subjected to multiple sequence alignment to find the conserved regions within the sequences. Figure 1 shows conserved regions at the N terminal of the sequences used to predict epitopes.

### 3.2. B Cell Epitopes

**Figure 2:**
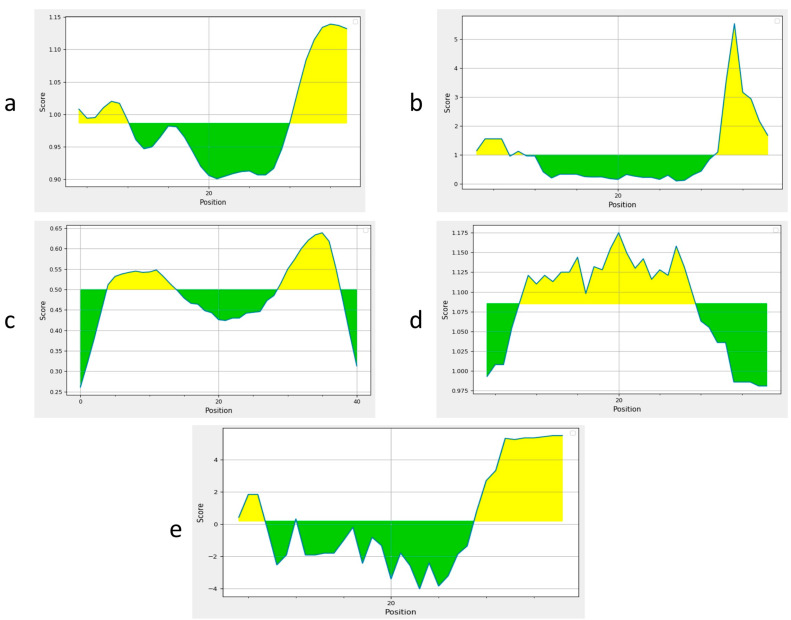
Prediction of B cell epitopes. Yellow-colored-regions above the threshold are supposed to be positive for B cell epitopes and the green-colored regions are supposed to be negative for B cell epitopes; (**a**) BepiPred analysis for B cell identification; (**b**) Emini Surface Accessibility Prediction; (**c**) Kolaskar and Tongaonker Antigenicity; (**d**) Karplus and Schulz flexibility to predict the flexibility; (**e**) The Parker Hydrophilicity.

Two B cell epitopes were identified using the IEDB server (Table 1). Epitopes with a length of 15mer and a threshold score of 0.9 were selected. Sequence analysis was performed. Predicted epitopes were antigenic, non-allergenic, and non-toxic so they met the criteria to construct a standard candidate vaccine design. These epitopes were further confirmed by using BepiPred2 and Emini Surface Accessibility, Kolaskar and Tongaonker, Karplus and Schulz flexibility, and Parker Hydrophilicity analyses, which further validated the B cell epitopes (Figure 2a–e).

### 3.3. Prediction of CTL Epitopes

Cytotoxic T lymphocytes are presented by MHC class I followed by CD8 T cell recognition. CTL epitopes were predicted by using the NetCTL 1.2 server. Only one epitope was predicted based on binding affinity (0.3701), cleavage (0.9251), and TAP (0.5170). Epitopes with good binding affinity can produce a high immune response. The predicted combined score was recorded as 0.5347 (Table 2).

### 3.4. Prediction of HTL Epitopes 

HTL epitopes are presented by MHC class II followed by the recognition of CD4 cells. These cells augment and intensify the immune response. The IEDB MHC II web server with default threshold values was used to predict HTL epitopes. Altogether, two HTL epitopes were predicted and shortlisted based on their antigenicity, non-allergenicity, and non-toxic nature, as shown in Table 3.

### 3.5. MEV Construct

**Figure 3:**
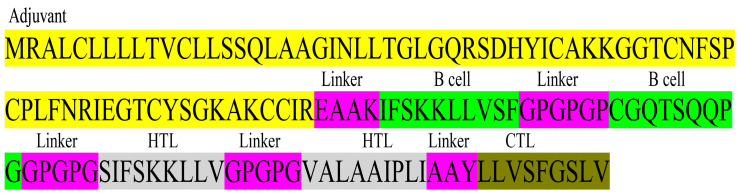
MEV construct design. MEV construct includes 138 amino acids comprising two B cell epitopes, two HTL epitopes, one CTL epitope, and an adjuvant ß-Defensin, all joined together by linkers.

The construct of the 138 total amino acids was designed by linking 2 B cell epitopes, 2 HTL, 1 CTL epitope, and an adjuvant (Figure 3). A sequence of ß-Defensin was assembled with the vaccine construct for enhancement of immunity. The prediction of the construct as a probable antigen with a score of 0.6876, non-allergenic, and non-toxic, indicates a good vaccine candidate.

### 3.6. Physicochemical Properties

Table 4 shows the physicochemical details of the designed vaccine candidate. The molecular weight is 14,153.88 Daltons with a theoretical isoelectric point (pI) of 9.42. An instability index of 26.5 shows the stable nature of the vaccine candidate. More positively charged residues were recorded as compared to negatively charged residues. The higher aliphatic index shows the tendency of protein adaptation at high temperatures. A Grand average of hydropathicity (GRAVY) score of 0.484 defines the polar and hydrophilic nature of the candidate protein. A scaled solubility of 0.707, higher than that of the average soluble protein of *E. coli,* indicates that the protein is highly soluble. All physicochemical properties of the MEV construct meet the criteria of a standard vaccine candidate. 

### 3.7. Secondary Structure and Solubility

**Figure 4:**
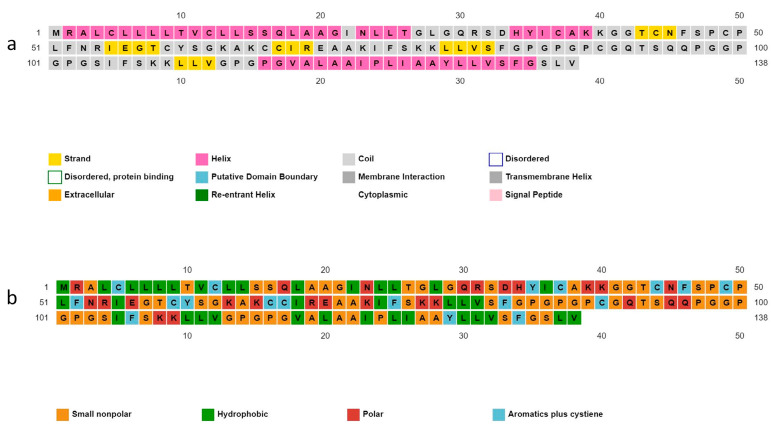
Predicted secondary structure of vaccine construct. (**a**) Structural residues are present in the form of strands, helix, and coil; (**b**) the nature of the residues as small non-polar, hydrophobic, polar, and aromatic.

Structural residues of the candidate protein are present in coil (51.4%), helix (36.2%), and strand (12.3%) forms. The solubility properties of the polypeptide backbone indicate the nature of the residues as small non-polar (42%), hydrophobic (25.3%), polar (18.1%), and aromatic plus cysteine (13.04%) (Figure 4).

### 3.8. Prediction and Refinement of Tertiary Structure

**Figure 5:**
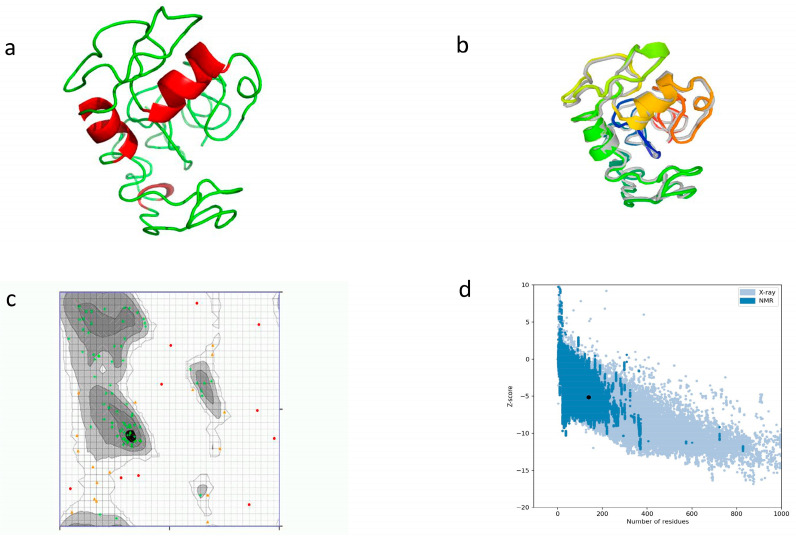
Prediction of tertiary structure with refinement and validation. (**a**) Tertiary structure model using I-TASSER; (**b**) refinement of tertiary structure by superimposition; and (**c**) Ramachandran plot for structure validation. Green crosses show highly preferred regions, brown triangles show preferred regions, and red dots are questionable; (**d**) structural quality analysis with Z-score.

By using I-TASSER, a three-dimensional tertiary structure was chosen, having a confidence score (C-score) of −3.62. For the aim of improving the quality of the model and its consistency, we used the GalaxyREFINE webserver. Among the five models generated, model 1 was selected depending on the factors related to the structure, including GDT-HA (0.8098), RMSD (0.734), and MolProbity (3.852). Other factors predicted included Clash score (107.3), rotamers (3.7), and Rama favored (58.8). The tertiary structure was validated by using a Ramachandran plot. It revealed that 74.528% (54.7% before refinement) of amino acids resided in highly preferred regions, while 16.038% (29.245% before refinement) of amino acids resided in preferred regions. We used the ProSA-web tool to validate the 3D model, and a Z-score of −5.12 was calculated (Figure 5).

### 3.9. Discontinuous B Cell Epitopes

**Figure 6:**
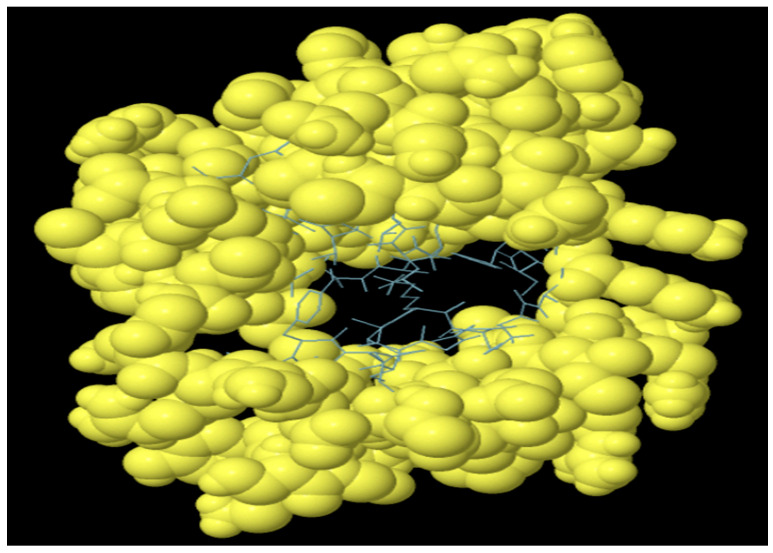
Predicted discontinuous B cell epitopes in MEV vaccine construct. Yellow balls show B cell epitopes and sticks represent the rest of polypeptide.

The antibodies produced by B-lymphocytes mediate the humoral immune response. Therefore, B cell epitopes are supposed to reside within the domain of the MEV construct. The vaccine construct harboring B cell epitopes is shown in Figure 6. A total of 104 residues with a score of 0.58 were predicted (Table 5).

### 3.10. Molecular Docking

**Figure 7:**
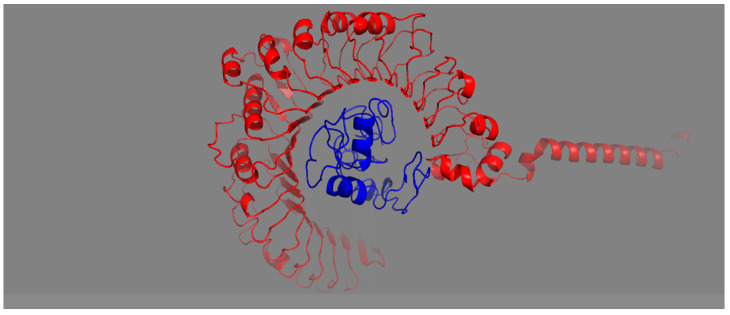
Structure docked with TLR2 receptor. Ligand–protein and receptor–protein are indicated by red and blue colors, respectively.

The affinity of the vaccine construct to the host Toll-like receptors indicates the intensity of immune stimulation. A vaccine construct having good binding with TLR-2 can stimulate a proper host immune response. The designed vaccine construct docked with TLR-2 receptors and showed strong affinity, indicating a robust adoptive and innate immunity response from host. Protein–protein docking was used to predict the interaction of MEV with the TLR2 immune receptor. The 3D structure of TLR2 receptors was retrieved from PDB, and molecular docking was performed. The server generated various docked structures, and the model was picked based on the lowermost intermolecular energy with high binding affinity throughout the docked structures. Moreover, the docked complex structure was analyzed and visualized using PyMOL (Figure 7).

### 3.11. Molecular Dynamics Simulation

**Figure 8:**
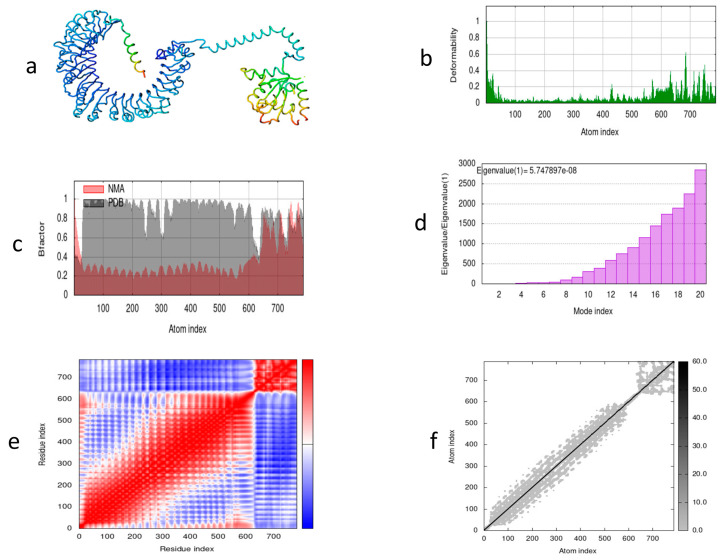
Molecular dynamics simulation of MEV candidate. (**a**) Vaccine structure after docking with TLR2; (**b**) deformability graph; (**c**) experimental B factors; (**d**) eigenvalues representing motion stiffness; (**e**) co-variance of docked vaccine representing red (correlated), blue (uncorrelated), and white (anti-correlated) regions; (**f**) elastic network with grey dots shows the level of stiffness.

Molecular dynamics was performed to determine the complex stability of the vaccine construct during simulation. The motion and stiffness of the predicted residues show that the vaccine construct is stable and suitable. Using the iMOD server, we illustrated the dynamics of atomic movement within the structure of the designed construct. Figure 8a shows the vaccine construct along with TLR2. Figure 8b illustrates the frequency of distortion within the structure. Figure 8c shows the NMA and PDB field relationship illustrated by the corresponding B factor. The motion of the structure shown by a eigenvalue of 5.747897 × 10^−8^ is illustrated in Figure 8d. Different kinds of coupling between pairs of residues are shown in Figure 8e, depicting correlated, anti-correlated, and uncorrelated. The stiffness of the structure is shown by the dots in Figure 8f; dark-grey-colored dots show the stiffness, while light-grey-colored dots show flexibility among the residues.

### 3.12. Molecular Cloning 

**Figure 9:**
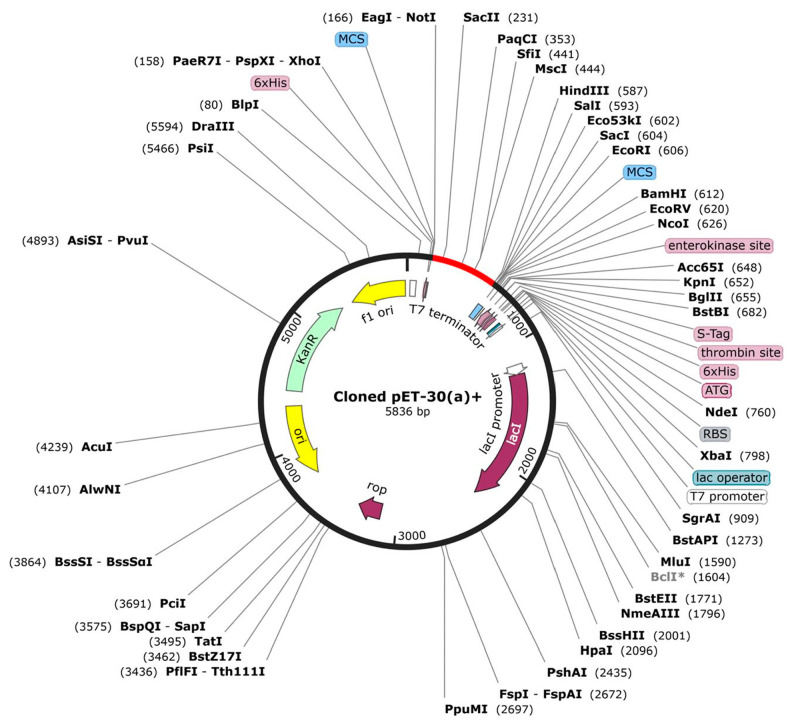
Proposed in silico cloning map of reverse-translated fragment of vaccine candidate by using free trial of SnapGene. Red fragment in the black backbone of the vector PET-30 (a) + shows inserted vaccine fragment.

The protein sequence was reverse-translated into a nucleotide sequence using the online web server JCat. The DNA sequence consisting of 5836 bp was improved by using a vector builder to obtain a codon optimization index of 0.93 and 59.9% GC content. Restriction sites for NotI and HindIII were inserted and cloned into PET-30 (a) + using a free trial of SnapGene as illustrated in Figure 9.

### 3.13. Immune Simulation

**Figure 10:**
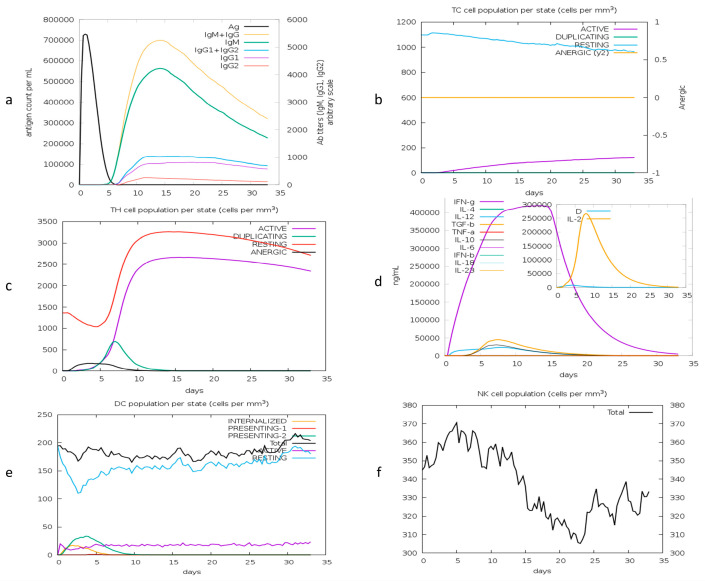
Computational immune response to antigen. (**a**) Production of immunoglobulins; (**b**) T helper cell population; (**c**) T cytotoxic cells; (**d**) production of interferons and interleukins after repeated injections; (**e**) dendritic cell population; (**f**) natural killer cells.

The immune simulation shows the host’s primary and secondary immune responses after injecting the vaccine. The immune profile confirms the production of antibodies and cytokines. The production of B and T (TH, TC) cells after injecting the antigen shows a highly responsive immune system. Cytokine production shows stimulated T-cell-mediated immunity in response to the vaccine. Figure 10a shows the primary response of antibodies, including IGM, IGM + IGG, and IGG1 + IGG2 responses. The antigen diminishes at day 6 post injection. Responses of T helper and cytotoxic cells in Figure 10b illustrate the developed memory in the host. The production of cytokines, including interleukins and interferons, is shown in Figure 10c. The response of dendritic cells (DCs) and natural killer cells (NK) is illustrated in Figure 10d,e, respectively. The predicted immune profile revealed that the proposed candidate vaccine can elicit an effective immune response.

## 4. Discussion

*M. hyorhinis* is known to cause several infections in swine and its pathogenesis has been poorly studied to date. Recent research work aided in a better understanding of the pathogenesis of this ubiquitous pathogen [5,27,28]. The use of antibiotics to treat infections leads to antimicrobial resistance, enhanced complications, and economic losses [29]. Vaccines can effectively induce a protective response in the host and hence help to reduce ailments and associated losses. Inactivated vaccines against *M. hyorhinis* have been developed and shown to be helpful in the reduction in lesions, resulting in the decline in infections [11,12,30]. An inactivated vaccine Ingelvac^®^ MycoMAX is being commercially used against swine diseases in some countries. Although inactivated vaccines produce a robust immune response, and there is a probability of poor efficacy towards non-vaccine strains, strict storage conditions and exposure to immunocompromised pigs during pregnancy or lactation make the vaccine compromised. A multi-epitope vaccine with animal models has shown advantages over the live or attenuated vaccine because of its efficacy, lower cost, and protection [31,32]. These vaccines produce robust immune responses, can be safely administered to the immunocompromised pigs, and do not require lower-temperature storage conditions [33]. Designing vaccines using immunoinformatics tools enables the identification of B and T cells that identify the fate of vaccine candidates [34]. Linear and conformational B cells produce antibodies while T cells take part in the recognition of surface antigens on the antigen-presenting cells (APCs). Selected B and T cell epitopes stimulate innate and adaptive immunity, resulting in protective memory. Recently, the application of computational methods for vaccine design has been the preferred approach by researchers [35,36,37,38]. The proposed candidate vaccine, by using a computational reverse vaccinology approach, can be evaluated through animal immunization assay and has proved to be an effective strategy. A study conducted by Li and colleagues used the computational approach to propose the vaccine candidate and successfully evaluated its efficacy in mice models [37]. Implementing a computational reverse vaccinology approach and its success in in vivo experiments are supported by several studies [39,40,41,42].

Variable lipoproteins are exposed on the pathogen’s surface, enabling them to directly interact with the host immune response and hence are highly antigenic. Despite the high degree of variability, these Vlps possess conserved domains that can be considered to serve as vaccine candidates. The antigenicity of VlpA-G of *M. hyorhinis* has evidence from previous research where unique repeated units in region III of VlpA-G were used to develop ELISA with remarkable specificity and sensitivity [7].

We assembled the conserved domains found at the N terminal of variable surface lipoproteins of *M. hyorhinis* to find CTL, HTL, and B cell epitopes. Epitopes were combined by using EAAK, GPGPGP, and AAY linkers that make the molecule more stable and prevent disruption [43]. Adding the ß Defensin adjuvant helps boost the immunogenicity of the vaccine candidate by enhancing the production of antibodies. It is an antimicrobial peptide that interacts with immune receptors, including TLRs and CCR6, and recruits naive T cells and immature DCs, stimulating an innate and adaptive immune response [44]. It has been previously used in various studies to design multi-epitope vaccines using the immunoinformatics approach [38,45]. The designed vaccine candidate is antigenic, non-allergenic, and non-toxic, fulfilling the basic requirements and standards as mentioned previously [46]. Physicochemical properties such as Theoretical PI, GRAVY, and stability index show that the proposed candidate vaccine is polar and stable. The results are similar to the previous studies and fulfill the criteria of a good vaccine construct [38,43]. The immune simulation showed a strong immune response with the production of IgM and IgG antibodies immediately after the elimination of the antigen. 

Lipoproteins on the membrane surface are critical antigens of *Mycoplasma* sp. Previous studies demonstrated the role of TLR2 in identifying *M. hyopneumoniae* in porcine alveolar macrophages, hence being the key factor to stimulate an innate immune response [47]. It has been shown that most bacterial lipoproteins induce an innate immune response through TLR2 receptors [48]. Moreover, many *Mycoplasma* sp. trigger an immune response via TLR2 receptors [49,50,51]. The involvement of TLR2 receptors in immune stimulation can be assessed by assessing the upregulation of interferons and a specific set of cytokines and immune regulatory factors such as NF-κB [52]. The designed vaccine construct was docked with TLR2, and it showed strong binding with TLR2 receptors, depicting a strong immune response.

## 5. Conclusions

*M. hyorhinis* is a pathogen identified in swine and is known to cause severe illness. Inactivated vaccines currently on the market have not been successful in controlling infections, because of certain limitations. By combining different epitopes, immunoinformatics has been hailed as a potentially fruitful method for developing vaccines. The conserved domains that can be found at the N terminal of VlpA-G in *M. hyorhinis* were utilized in our research. The MHC-I, MHC-II, and B cell epitopes, in addition to linkers and an adjuvant, were utilized to propose a probable multi-epitope candidate vaccine. The designed proposed vaccine construct was antigenic, did not contain any allergens, and demonstrated excellent stability and binding ability to TLR2 receptors. In addition, the candidate vaccine elicited a robust immune response from both the humoral and cell-mediated types. To validate its safety and efficacy, additional experiments, both in vitro and in vivo, need to be conducted.

## Figures and Tables

**Table 1:** Predicted B cell epitopes in the conserved sequences Vlps.

No.	Start	End	Peptide	Length
1	5	14	IFSKKLLVSF	10
2	30	38	CGQTSQQPG	9

**Table 2:** Predicted CTL epitopes in the conserved domains of VlPs.

Peptide Sequence	Binding Affinity	Affinity Rescaled	Cleavage	TAP	Combined Score
LLVSFGSLV	0.0872	0.3701	0.9251	0.5170	0.5347

**Table 3:** Predicted HTL epitopes in the conserved sequences of Vlps.

Peptide Sequence	Start	End	Score
SIFSKKLLV	4	12	0.194
VALAAIPLI	18	26	0.24

**Table 4:** Physicochemical properties of vaccine candidate.

Physicochemical Properties	Score
Number of amino acids	138
Molecular weight	14,153.88
Theoretical pI	9.42
Total number of negatively charged residues (Asp + Glu)	3
Total number of positively charged residues (Arg + Lys)	13
Ext. coefficient	4970
Estimated half-life	>20 h (yeast, in vivo) >10 h (*Escherichia coli*, in vivo)
Instability index (II)	26.53 (Stable)
Aliphatic index	103.26
Grand average of hydropathicity (GRAVY)	0.484 (Polar protein)
Scaled solubility	0.707
Number of amino acids	138

**Table 5:** Predicted discontinuous epitopes in multiepitope vaccine construct by using ElliPro.

No.	Residues	Number of Residues	Score
1	A:M1, A:R2, A:A3, A:L4, A:C5, A:L6, A:L7, A:L8, A:L9, A:T10, A:V11, A:L14, A:S15, A:S16, A:Q17, A:L18, A:A20, A:G21, A:L24, A:L25, A:T26, A:G27, A:L28, A:G29, A:Q30, A:R31, A:S32, A:D33, A:C44, A:N45, A:F46, A:S47, A:P48, A:C49, A:P50, A:L51, A:F52, A:N53, A:R54, A:I55, A:E56, A:G57, A:T58, A:C59, A:Y60, A:S61, A:G62, A:K63, A:A64, A:K65, A:C66, A:C67, A:I68, A:R69, A:E70, A:K73, A:K77, A:L80, A:V81, A:S82, A:F83, A:G84, A:P85, A:G86, A:P87, A:G88, A:P89, A:C90, A:G91, A:Q92, A:T93, A:S94, A:Q95, A:Q96, A:P97, A:G98, A:G99, A:P100, A:G101, A:P102, A:G103, A:S104, A:I105, A:F106, A:S107, A:K108, A:K109, A:V112, A:G113, A:P114, A:G115, A:P116, A:A121, A:A122, A:I123, A:P124, A:L125, A:I126, A:S133, A:F134, A:G135, A:S136, A:L137, A:V138	104	0.58

## Data Availability

The data presented in this study are available within the article.
